# Supplementary material for: Caenorhabditis elegans RMI2 functional homolog-2 (RMIF-2) and RMI1 (RMH-1) have both overlapping and distinct meiotic functions within the BTR complex
Source: PLoS Genet. 2021 Jul 12;17(7):e1009663. doi: 10.1371/journal.pgen.1009663 (PMC8318279; doi:10.1371/journal.pgen.1009663)
Supplement: Attachment — Submitted filename: response to reviewers.pdf [file pgen.1009663.s008.pdf]

**We again want to thank all reviewers for their time and efforts to improve the manuscript.**

Reviewer #1: The revised manuscript by Velkova et al., describes the identification and functional characterization of the C. elegans ortholog of RMI2 (RMIF-2), a member of the BTR complex critical for recombination processing. The authors have done a good job addressing the reviewers' comments, including addition of statistics and additional analyses of the rmif-2 rmh-1 double mutant. Overall, this is an important study that warrants publication in PlosGenetics.

I only have a couple of very minor changes:

1. Line 40: remove the last "the"

***done***

2. Line 276: I think you mean rmif-2 (not rmh-1).

***No, actually we talk about rmh-1 and in this study we examined COSA-1 in rmif-2 in addition. Since we used a new tagged line of COSA-1, we also repeated the analysis in rmh-1.***

3. Line 315: please add: "as" depicted

***done***

4. Line 353-354: For me the "mitotic" failure came out of nowhere – what is the evidence that it is both meiotic and mitotic failure? Please clarify.

***We took this sentence out. It should not be here.***

5. Line 365: remove "the" meiotic DSB repair

***done***

Reviewer #2: I appreciate the improvements made in the manuscript, especially with regard to improved precision of the text, and some added statistical analysis. I also appreciate the added data regarding the unexpected (lack of) MSH-5 localization in the rmh-1 mutant. That said, my fundamental concerns about the manuscript remain, and I don't find the manuscript 'much-revised'. Most notably, I think the manuscript still lacks mechanistic insight into meiotic functions of the BTR complex, and into the specific contributions of RMIF-2 to those functions. That issue was not addressed to a significant level in this revision, and remains the main hurdle for publishing this paper. Several other more technical points:

While the clarifications on MSH-5 localization validate the technical aspects of the experiment, the issue still remains mysterious. As the results stand, they suggest that we need to examine what do MSH-5 foci mean at all, since MSH-5 seem to be able to act without making foci. (Related argument could be made regarding the faint and bright HIM-6 foci, the functional meaning of which remains unknown).

***This is a very important point that was made here: what do foci actually mean? We have indeed made a statement about activities that might take place outside countable foci in the discussion.***

***For the MSH-5: we have added the following sentence:*** **It seems as if MSH-5 is operating at recombination intermediates in the *rmh-1* mutant, but the levels are too low for detection or MSH-5 activity is not always found in foci.**

The western blots and their quantification remain confusing. For example, in Figure 1B, why is there such a difference between the HA levels in the two genotypes in the 10% input part?

***We have really given our best to detect those proteins, with the reagents available. We tested many different reagents to do HA and FLAG-W-blots. The reagents we used here in the end, were the best ones we could find. It is not trivial to do biochemistry with recombination proteins from C. elegans gonads (due to the low amounts of those proteins in the extracts). Figure 1B is meant to make the point: RMIF-2***

***pulls down RMH-1 to substantiate the mass spec results. Mass spectrometry with C. elegans gonads is also not at all trivial; in fact, with RMH-1 pull downs we managed to reproducibly pull down peptides encoded by the novel ORF (later named RMIF-2) in triplicates. The W-blots are meant to substantiate the mass spec data. As reviewer 2 wished, we marked unspecific bands with * and **. We really would not know what to add to the text.***

In Figure 3B-C, there is clearly a band in the HA blot in the ha::rmh-1 rmif-2 genotype, but it is quantified as zero.

***The quantification is derived from three representative blots where we normalize the protein quantity of ha::rmh-1 vs the LMN-1 control. This is stated in the legend.***

There are still unsupported claims regrading the IF data (meaning unquantified or not-statistically validated). Claims relating to intensity of foci or signal should be more robustly quantified or removed. The authors' rebuttal is correct - these are difficult experiments. But without more robust controls and quantifications, the text should be further clarified to indicate the impressionistic nature of the observations.

***We deleted the HIM-6 foci quantification in S3 Fig3 (together with the corresponding parts in the legend and methods). The quantification was meant to underscore the "different quality" of the foci we see—but this can also just be seen in the image.***

Reviewer #3: The authors addressed all my concerns

I have a couple of minor suggestions (not required)
Line 357- I suggest refraining from using the term "non-significant increase", as if it's not significant, it's not an increase, it should just say that there is no effect/change/increase.

***Done***

Figure 8B- I'm happy the significance was added with Fisher's exact test, but it still looks strange to have a single data point in a graph (consider adding to table instead?). I believe the same issue is in 7B.

***We have now modified the graph from figure 8C and removed the single data point as we want to present the percentage of heterologous recombination in the different genotypes. In the table in figure 8B one can find the raw data of recombinants observed verses the total amount of animals analyzed.***

***Figure 7: we believe that showing the percentage of the recombination frequency along the length of the chromosomes with specific genetic intervals is a valuable way to compare the theoretical, wild type and mutant recombination landscapes. These values represent the percentage of recombination frequency obtained from 200-400 worms for each of the analyzed chromosomes in each specific genetic interval AB, BC, CD, DE.***
